# Emodin from Aloe inhibits Swine acute diarrhea syndrome coronavirus in cell culture

**DOI:** 10.3389/fvets.2022.978453

**Published:** 2022-08-18

**Authors:** Shumei Zheng, Xiaowei Wang, Huiqiong Hu, Yongbo Xia, Xiaoyuan Diao, Wenjing Qiu, Chunyi Xue, Yongchang Cao, Zhichao Xu

**Affiliations:** State Key Laboratory of Biocontrol, School of Life Science, Sun Yat-sen University, Guangzhou, China

**Keywords:** Emodin, swine acute diarrhea syndrome coronavirus (SADS-CoV), antiviral activity, virus attachment, toll-like receptor 3 (TLR3), IFN-λ3, ISG15

## Abstract

Swine acute diarrhea syndrome coronavirus (SADS-CoV) is an emerging swine enteropathogenic coronavirus that causes severe diarrhea in neonatal piglets, leading to serious economic losses to the pig industries. At present, there are no effective control measures for SADS, making an urgent need to exploit effective antiviral therapies. Here, we confirmed that Aloe extract (Ae) can strongly inhibit SADS-CoV in Vero and IPI-FX cells *in vitro*. Furthermore, we detected that Emodin from Ae had anti-SADS-CoV activity in cells but did not impair SADS-CoV infectivity directly. The time-of-addition assay showed that Emodin inhibits SADS-CoV infection at the whole stages of the viral replication cycle. Notably, we found that Emodin can significantly reduce virus particles attaching to the cell surface and induce TLR3 (*p* < 0.001), IFN-λ3 (*p* < 0.01), and ISG15 (*p* < 0.01) expressions in IPI-FX cells, indicating that the anti-SADS-CoV activity of Emodin might be due to blocking viral attachment and the activation of TLR3-IFN-λ3-ISG15 signaling axis. These results suggest that Emodin has the potential value for the development of anti-SADS-CoV drugs.

## Introduction

Swine acute diarrhea syndrome coronavirus (SADS-CoV), also named porcine enteric alphacoronavirus (PEAV) (1) and swine enteric alphacoronavirus (SeACoV) (2), is a novel porcine coronavirus that belongs to the genus Alphacoronavirus of the family Coronaviridae (3) together with transmissible gastroenteritis virus (TGEV) (4) and porcine epidemic diarrhea virus (PEDV) (5). SADS-CoV is an enveloped, positive-sense, single-stranded RNA virus (3). Its full-length genome is about 27 kb and arranged in the order of 5′UTR-ORF1a/1b-S-NS3-E-M-N-NS7a-NS7b-3′UTR, encoding 16 non-structural proteins, 3 accessory proteins, and 4 structural proteins (3). The clinical signs caused by SADS-CoV are similar to other porcine enteric pathogens, such as TGEV and PEDV, which include acute vomiting, watery diarrhea, and dehydration. The mortality rate in <5 days old piglets was as high as 90%, whereas it dropped to 5% in piglets that were older than 8 days (3). SADS-CoV was first detected in pig herds with diarrhea outbreak in Guangdong in 2017 (1) and the retrospective study confirmed that SADS-CoV appeared in China at least in August 2016 (6). Even though there were no new SADS cases reported in pigs in Guangdong from May 2017 to January 2019, the re-emerging of SADS-CoV infection in pig herds in southern China in February 2019 (7) indicated a continuing threat of SADS-CoV to the pig farms. Apart from Guangdong, a SADS-CoV strain, CN/FJWT/2018, was discovered in Fujian, China (8). Of note, SADS-CoV has recently been found to infect a variety of human cell lines (9, 10), indicating that SADS-CoV might be a potential higher-risk coronavirus pathogen to impact human health. Given the great harm of severe acute respiratory syndrome coronavirus 2 (SARS-CoV-2) (11), the research and development of SADS-CoV of prevention and control measures might have important public health significance.

To combat the virus, antiviral therapy, one of the present management strategies, is the option for the control of the SADS-CoV infection. At present, there are no effective control measures for SADS, making an urgent need to exploit effective antiviral therapies. Natural products are considered an important source of new generations of antiviral agents (12). Aloe vera, a common plant, has a broad-spectrum antiviral activity against both DNA and RNA viruses, such as influenza virus, herpes simplex virus type 1, pigeon paramyxovirus type 1, porcine reproductive and respiratory syndrome virus (PRRSV), and PEDV (13–17). Antiviral effects have been confirmed not only for the whole extracts of Aloe but also for a variety of active chemical ingredients it contains. Emodin (1,3,8-trihydroxy-6-methylanthraquinone; C_15_H_10_O_5_) is a natural bioactive anthraquinone with conjugated double bonds extracted from the roots and bark of Rhubard, Aloe, and other medicinal plants (18). Some studies have shown that Emodin has antiviral activity against coxsackievirus B3 (19), enterovirus 71 (20), herpes simplex virus (21), human coronavirus OC43 (HCoV-OC43) (22), SARS-CoV (23), SARS-CoV-2 (24), and PRRSV (17), which is achieved by blocking the virus–receptor interaction (23), restraining the M^pro^ activity (24), and inhibiting the translation of viral proteins (19), viral maturation (20), and the release of the virus (22). In addition, it has been reported that Emodin can also activate the host's innate immunity and inhibits PRRSV replication by activating the TLR3-IFN-α pathway (17).

Although Aloe and its component Emodin have demonstrated antiviral activity against many viruses, detailed information about the antiviral effect of SADS-CoV remains unclear. In this study, the antiviral activity of Aloe on SADS-CoV was first assessed *in vitro*. Furthermore, we examined the anti-SADS-CoV activity of Emodin from Aloe *in vitro* and identified the stages of the SADS-CoV life cycle that Emodin might target. Finally, we elucidated the potential mechanism of Emodin's anti-SADS-CoV activity. Our results showed that Emodin from Aloe can effectively inhibit SADS-CoV replication *in vitro*, which might mainly involve with blocking viral attachment and activating the TLR3-IFN-λ3-ISG15 pathway, indicating that Emodin has the potential value as a candidate drug against SADS-CoV.

## Materials and methods

### Cells, virus, and virus preparation

Vero cells (ATCC number: CCL-81) were obtained from ATCC (USA) and IPI-FX cell lines were kindly provided by Professor Shaobo Xiao (Huazhong Agricultural University, Wuhan, China) (25). Both Vero and IPI-FX cells were cultured in Dulbecco's modified eagle medium (DMEM) (Gibco, Scotland, UK) supplemented with 10% fetal bovine serum (FBS) (Gibco, Scotland, UK), 100 U/ml penicillin, and 100 U/ml streptomycin. All cells were cultured in an incubator at 37 °C with 5% CO_2_. The maintenance medium was serum-free DMEM supplemented with 10 μg/ml trypsin for Vero cells or 1 μg/ml trypsin for IPI-FX cells.

The SADS-CoV GDS04 strain was isolated from piglets with severe diarrhea in our laboratory (26) and propagated in Vero cells as previously described (26). The cell lysates and supernatant samples harvested together were subjected to viral titers using the 50% tissue culture infective dose (TCID_50_) assay. Briefly, Vero cells were seeded in 96-well plates and were grown as adherent monolayers. The cells were washed with 1 × phosphate-buffered saline (PBS) three times, 100 μl of 10-fold serial dilutions of virus-containing samples were added to each well, and then the cells were continuously cultured at 37 °C in 5% CO_2_. The cytopathic effect (CPE) was observed for 5–7 days, and virus titers were calculated by the Reed–Muench method (27) and expressed as the TCID_50_. The plaque forming unit (PFU) was calculated by the following equation: PFU = 0.7 × TCID_50_ (28), and PFU was used to determine the multiplicity of infection (MOI).

### Cell viability assay

The cytotoxicity of drugs to Vero and IPI-FX cells was measured by the Cell Counting Kit-8 (CCK-8) (Yeasen Biotech, Shanghai, China) according to the manufacturer's instructions. Briefly, Ae (Bioforte Biotechnology Co., Ltd., Shenzhen, China) was dissolved in DMEM at a concentration of 100 mg/ml. Aloin (Solarbio, Beijing, China), Quercetin (Solarbio, Beijing, China), and Emodin (Sigma-Aldrich, Shanghai, China) were dissolved in dimethyl sulfoxide (DMSO) at concentrations of 400 μg/ml, 100 μg/ml, and 400 μg/ml, respectively. Vero and IPI-FX cells were seeded in 96-well plates and cultured for 90% confluence. The cells were treated with serial dilution of drugs (Ae, Aloin, Emodin, and Quercetin) or the normal culture medium or the culture medium containing 0.1% DMSO. After incubation for 24 h and 48 h, 100 μl of culture medium containing 10% CCK-8 solution was added to each well and reacted for 1 h at 37 °C, and the absorbance was measured at 450 nm using a microplate reader The relative viability of cells was calculated by “cell viability (%) = [OD_450nm_ (drugs)–OD_450nm_ (blank) / OD_450nm_ (controls)–OD_450nm_ (blank)] × 100%.”

### Inhibition of SADS-CoV infection assay

Vero or IPI-FX cells were seeded in 12-well plates and cultured overnight. The cells were exposed to different concentrations of drugs (Ae, Aloin, Emodin, and Quercetin) or the normal maintenance medium or the maintenance medium containing 0.1% DMSO for 1 h before SADS-CoV infection. One hour after SADS-CoV infection at an MOI of 0.1, the viral inoculums were changed and the cells were treated with indicated doses of drugs again. At the indicated time points (12 h, 24 h, and 48 h), cells were collected and cell lysates were prepared. Indirect immunofluorescence assay (IFA), Western blot, and TCID_50_ assay were performed to determine the antiviral activity.

### Indirect immunofluorescence assay (IFA)

Vero or IPI-FX cells infected with SADS-CoV were observed by IFA as described previously with some modifications (26). In brief, SADS-CoV-infected cells were fixed with 4% paraformaldehyde for 15 min and then permeabilized with 0.5% (w/v) Triton X-100 for 15 min at room temperature. After blocking with 3% bovine serum albumin (BSA) for 1 h, the cells were incubated with mouse polyclonal antibody against SADS-CoV N protein (1:1,000) and Cy3-labeled sheep anti-mouse secondary antibody (Proteintech Group, Inc., Chicago, IL, USA) at 37 °C for 1 h. After washing 3 times in 1 × PBS, the cell nuclei were counterstained with 4,6-diamidino-2-phenylindole (DAPI) (Beyotime, Shanghai, China). The Fluorescence microscope (NIKON Eclipse 80i, Tokyo, Japan) was used to observe the immunofluorescence.

### Western blot analysis

The cell samples were fully lysed in RIPA lysis buffer (Beyotime, Shanghai, China) containing 1% protease inhibitors (Yatai Hengxin, Beijing, China). The supernatants were collected by centrifugation at 4 °C and boiled with 5 × sodium dodecyl sulfate (SDS) loading buffer (Fdbio Science, Hangzhou, China) for 10 min. The protein samples were separated by 12% SDS-polyacrylamide gels (SDS-PAGE) electrophoresis and then transferred to the polyvinylidene fluoride (PVDF) membrane (Millipore, New Jersey, USA). After blocking with 4% BSA, the membranes were incubated with anti-SADS-CoV N polyclonal antibody (1:1,000) and anti-GAPDH antibody (Proteintech Group, Inc., Chicago, IL, USA) (1:3,000) at 4 °C overnight. Subsequently, the membrane was washed with 1 × Tris-buffered saline Tween 20 (TBST) buffer three times and incubated with horseradish peroxidase (HRP)-conjugated goat anti-mouse (1:5,000) (Proteintech Group, Inc., Chicago, Illinois, USA) or HRP-conjugated goat anti-rabbit IgG (1:5,000) (Proteintech Group, Inc., Chicago, Illinois, USA) at room temperature for 1 h. The blots were detected with the enhanced chemiluminescent (ECL) reagent (NCM Biotech, Suzhou, China).

### The assay of direct virion inactivation activity of Emodin

The effect of Emodin to inactivate SADS-CoV was directly determined, as described previously (17). Briefly, 1 × 10^5^ PFU of SADS-CoV GDS04 was mixed with 12.5 μg/ml Emodin at 37 °C for 1 h and 3 h. After drug treatment, the TCID_50_ assay was performed as described earlier to determine the virus infectivity of the samples.

### Time course analysis

The confluent monolayer of IPI-FX cells in 12-well plates was incubated with SADS-CoV at an MOI of 0.1 at 4 °C for 1 h for simultaneous infection. After removing the inoculum, 1 ml of maintenance medium was added to each well and continued incubation at 37 °C. Emodin solution was added to the wells or the viral samples to the final concentration of 12.5 μg/ml at various time points (**Figure 4A**). After 24 h, cells were collected and the mRNA and protein levels of SADS-CoV N in the cells were detected by quantitative real-time PCR (qRT-PCR) as described below and Western blot as described above, respectively. Virus titers in the cell lysates were determined by the TCID_50_ assay, as described above.

### Viral attachment assay

IPI-FX cells were seeded on coverslips (Biosharp, Anhui, China) before inoculation with SADS-CoV at an MOI of 5 for 2 h at 4 °C together with Emodin (12.5 μg/ml) or 0.1% DMSO. Coverslips were washed with 1 × PBS three times to remove unbound viruses and the cells were fixed with 4% paraformaldehyde at room temperature for 15 min. The following procedure was the same as IFA, as described earlier. Confocal images were examined using a confocal microscope (Leica TCS SP8 STED 3X, Berlin, Germany) equipped with a 100 × NA oil-immersion objective.

### Activation of the antiviral innate immune response by Emodin after SADS-CoV infection

IPI-FX cells were raised in 12-well plates for 12 h before treatment with Emodin (12.5 μg/ml) or 0.1% DMSO for 1 h and then infected with SADS-CoV at an MOI of 0.1. One and a half hours after infection, the viral inoculums were removed and fresh maintenance mediums containing Emodin (12.5 μg/ml) or 0.1% DMSO were added again. Only 0.1% DMSO or Emodin (12.5 μg/ml) or SADS-CoV were added as controls. After 12 or 24 h, total RNA was extracted for cDNA synthesis, and the quantitative real-time PCR assay was performed to examine the mRNA expression levels of pig TLR3, IFN-α, IFN-β, IFN-λ1, IFN-λ3, ISG15, and GAPDH, as described below.

### RNA extraction and quantitative real-time PCR

Total RNA was extracted from IPI-FX cells using the EZ-press RNA Purification Kit (EZBioscience, Roseville, MN, USA) and 450 ng RNA was reversely transcribed into cDNA using the RT-PCR Kit (TaKaRa, Dalian, China) according to manufacturer's instructions. The sequences of the specific primers are listed in Table 1 (29–31). The quantitative real-time PCR assay was performed by a Light Cycler 480 (Roche, Basel, Switzerland) and each PCR reaction was carried out in a 10-μl volume containing 1 μl of cDNA, 5 μl 2 × PerfectStart™ Green qPCR SuperMix (TransGen Biotech, Beijing, China), and 0.2 μM of each gene-specific primer. The thermal cycling parameters were as follows: 94 °C for 5 min; 40 cycles of 94 °C for 10 s, 58 °C for 20 s, and 72 °C for 30 s; and 1 cycle of 95 °C for 5 s, 65 °C for 1 min, and 95 °C for 15 s. The final step was to obtain a melt curve for the PCR products to determine the specificity of the amplification. All samples were tested in triplicate on the same plate, and the amplified products were calculated using the comparative threshold cycle (Ct) method. The mRNA expression levels of *N, TLR3, IFN-*α, *IFN-*β, *IFN-*λ*1, IFN-*λ*3*, and *ISG15* genes were normalized to the expression of the *GAPDH* gene.

**Table 1:** The primers used for qRT-PCR in this study.

**Primer**	**Sequence**
SADS-CoV F	5′-CTGACTGTTGTTGAGGTTAC-3′
SADS-CoV R	5′-TCTGCCAAAGCTTGTTTAAC-3′
GAPDH F	5′-CCTTCCGTGTCCCTACTGCCAAC-3′
GAPDH R	5′-GACGCCTGCTTCACCACCTTCT-3′
TLR3 F	5′-TAACAACCTTCCAGGCATA-3
TLR3 R	5′-AAGAGGAGAATCAGCGAGTG-3
IFN-α F	5′-TCTCATGCACCAGAGCCA-3′
IFN-α R	5′-CCTGGACCACAGAAGGGA-3′
IFN-β F	5′-AGTGCATCCTCCAAATCGCT-3′
IFN-β R	5′-GCTCATGGAAAGAGCTGTGGT-3′
IFN-λ1 F	5′-ATGGCTACAGCTTGGATCGTGGTG-3′
IFN-λ1 R	5′-GAGGGGAGAGCTGCAGCTCC-3′
IFN-λ3 F	5′-CCTTCAAGAGGGCCAAGGATGCC-3′
IFN-λ3 R	5′-GTGAAGGGGCTGGTCCAGGC-3′
ISG15 F	5′-AGCATGGTCCTGTTGATGGTG-3′
ISG15 R	5′-CAGAAATGGTCAGCTTGCACG-3′

F, forward; R, reverse.

### Statistical analysis

Results were analyzed using the GraphPad Prism software 5.0 (GraphPad, San Diego, CA, USA), and groups (cell viability, PFU, N mRNA, TLR3, IFN-α, IFN-β, IFN-λ1, IFN-λ3, and ISG15) were compared using ANOVA and Mann–Whitney tests, accordingly. *P*-value of < 0.05 was considered statistically significant.

## Results

### Ae inhibits SADS-CoV infection *in vitro*

**Figure 1:**
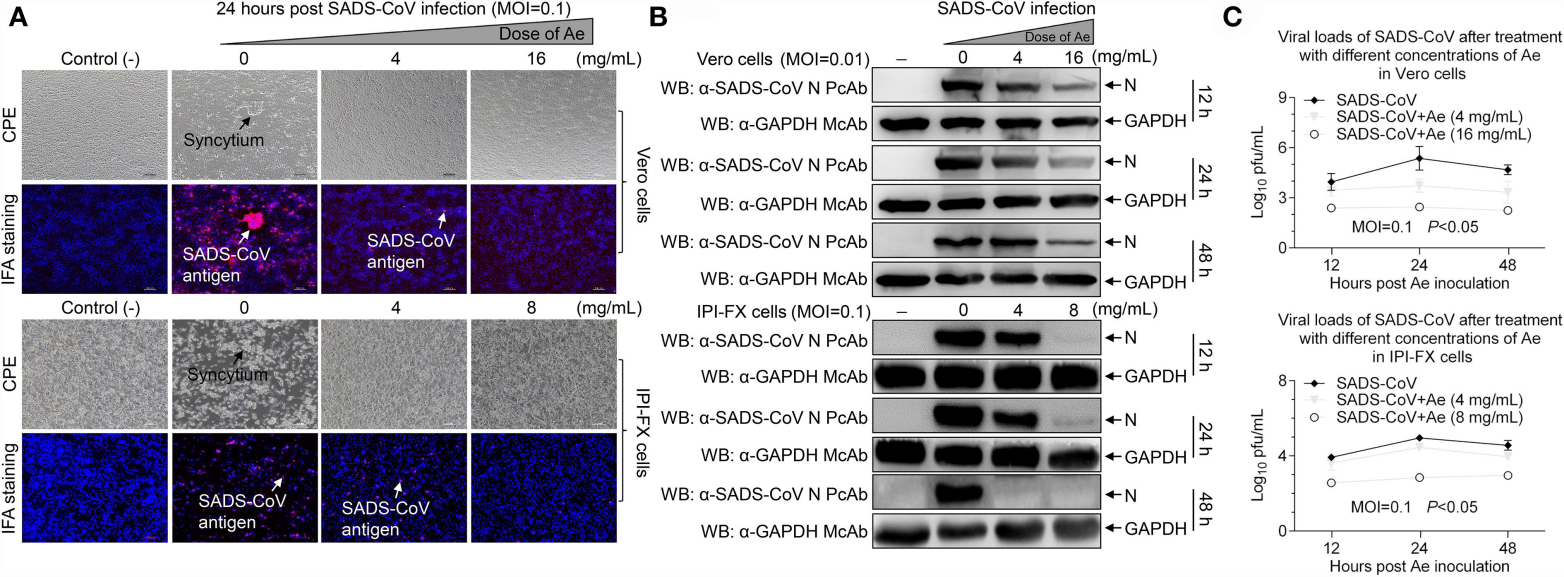
The effect of Ae on SADS-CoV infectivity. **(A)** Vero and IPI-FX cells were pre-incubated with different concentrations of Ae (2–16 mg/ml) for 1 h, followed by infection with SADS-CoV at an MOI of 0.1. After 1.5 h, the cells were re-treated with Ae or the normal medium. The positive red IFA signals for SADS-CoV N protein were monitored by fluorescence microscopy at 24 hpi. CPE and SADS-CoV antigens were indicated by arrows. Vero and IPI-FX cells were treated as above described, cell lysates were prepared at indicated time points (12 h, 24 h, and 48 h), and the expression level of N and GAPDH proteins was examined by Western blot using anti-SADS-CoV N polyclonal antibody and anti-GAPDH monoclonal antibody **(B)**, or viral titers were determined by the TCID_50_ assay **(C)**. Results are representative of three independent experiments (mean ± SD). *n* = 3.

To determine the effect of Ae on cell viability, the CCK-8 assay was used to detect the cytotoxicity of Ae at various concentrations. As shown in Supplementary Figure 1, after the cells were co-incubated with 0–32 mg/ml Ae for 24 h and 48 h, the relative cell viability was calculated. It was found that compared with the control group, the cell viability of Vero cells treated with 2–16 mg/ml Ae was 100%. However, the cell viability of Vero cells was reduced to 50% (*p* < 0.001) when the concentration of Ae was 32 mg/ml. Interestingly, the cell viability of IPI-FX cells treated with 2–8 mg/ml Ae was 100%, and then dropped to 20% as the concentration was 16 mg/ml (*p* < 0.001), indicating that Ae has different cytotoxicity in different cells. Based on the safe concentration of Ae, we examined the inhibitory effect of Ae against SADS-CoV using IFA. As indicated in Figure 1A, with the concentration of Ae continued to increase, the SADS-CoV-specific immunofluorescence in the infected cells was weakened. To further determine the anti-SADS-CoV activity of Ae, Western blot was used to detect the expression of N and GAPDH protein in Vero or IPI-FX cells at 12 hours post-infection (hpi), 24 hpi, and 48 hpi. The expression levels of N protein in the infected cells gradually decreased as the concentration of Ae increased, indicating that the inhibition of SADS-CoV by Ae was dose-dependent (Figure 1B). In addition, we used the TCID_50_ assay to detect the virus titers after treatments with Ae. The virus titers were significantly reduced in the high concentration group compared with the control group (*p* < *0.05*) (Figure 1C). Taken together, these data suggested that Ae can inhibit SADS-CoV replication *in vitro*.

### Emodin from Ae inhibits SADS-CoV replication *in vitro*

**Figure 2:**
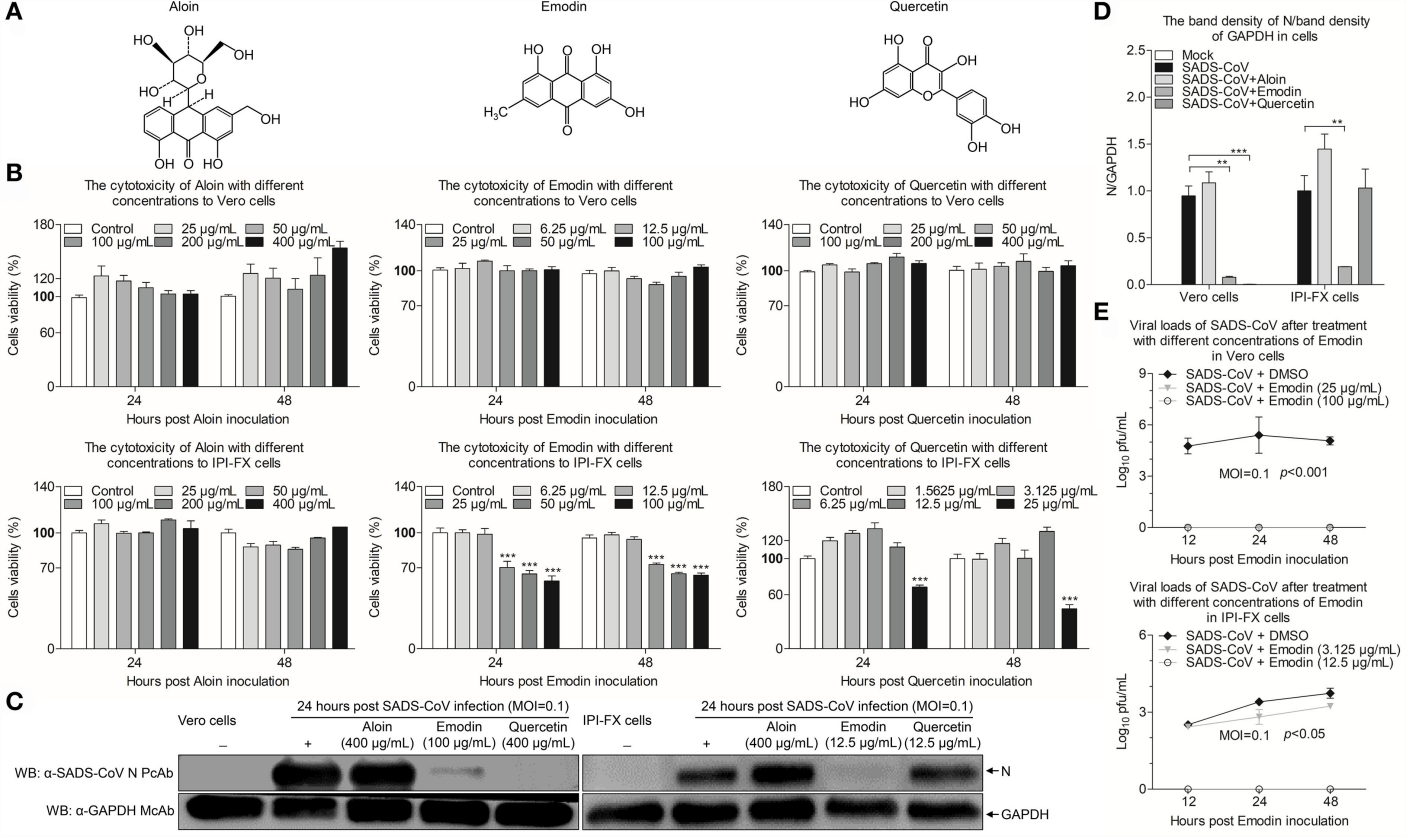
Emodin from Ae inhibits SADS-CoV replication *in vitro*. **(A)** Chemical structures of Aloin, Emodin, and Quercetin. **(B)** The cytotoxicity of Aloin, Emodin, and Quercetin in Vero and IPI-FX cells. Vero and IPI-FX cells were co-incubated with various concentrations of Aloin (25–400 μg/ml), Emodin (6.25–100 μg/ml), and Quercetin (1.5625–400 μg/ml) or 0.1% DMSO for 24 h and 48 h prior to the CCK-8 assay. **(C)** Vero and IPI-FX cells were pre-incubated with Aloin, Emodin, and Quercetin, or 0.1% DMSO at a safe concentration for 1 h, followed by infection with SADS-CoV at an MOI of 0.1. After 1.5 h, the cells were re-treated with Aloin, Emodin, and Quercetin or the normal medium. At 24 h post-inoculation, the expression levels of N and GAPDH proteins in the cell lysates were detected by Western blot using anti-SADS-CoV N polyclonal antibody and anti-GAPDH monoclonal antibody. **(D)** The relative quantity of SADS-CoV N protein described in **(C)**. **(E)** At indicated time points (12 h, 24 h, and 48 h), the viral titers in the cell lysates were determined by the TCID_50_ assay. Results are representative of three independent experiments (mean ± SD). *n* = 8 or 3. **p < 0.01, ***p < 0.001.

Aloin, Emodin, and Quercetin have been identified in the extracts of Aloe (Figure 2A) (17). To determine whether these components from Ae have the anti-SADS-CoV effects, initially, cytotoxicity of Aloin, Emodin, and Quercetin was rated by the CCK-8 assay. As shown in Figure 2B, the cell viability of Vero and IPI-FX cells by treatment with 400 μg/ml Aloin was 100%. The safe concentrations of Emodin and Quercetin to Vero cells are 100 μg/ml and 400 μg/ml, respectively. Interestingly, the safe concentrations of Emodin and Quercetin in IPI-FX cells are 12.5 μg/ml. Western blot was used to further investigate whether these components have anti-SADS-CoV activities. As shown in Figures 2C,D, the expression of N protein did not decrease in the infected cells treated with Aloin, indicating Aloin did not have anti-SADS-CoV activity. The N protein levels in the Vero cells treated with Quercetin significantly reduced (*p* < 0.001), but in IPI-FX cells did not decline dramatically, indicating that Quercetin did not have anti-SADS-CoV activity when the concentration dropped to 12.5 μg/ml and below. Of note, the N protein levels in the Emodin-treated infected cells were lower than that in the non-drug-treated infected cells (*p* < 0.01), indicating that Emodin has the best anti-SADS-CoV activity among the three components. To evaluate the influence of Emodin on viral infectivity, the TCID_50_ assay was used to detect the SADS-CoV titers after Emodin treatment. The virus titers significantly decreased after Emodin treatment (*p* < 0.05) (Figure 2E), as compared to the control. Collectively, Emodin from Ae has an anti-SADS-CoV activity.

### Emodin cannot directly impair SADS-CoV infectivity

**Figure 3:**
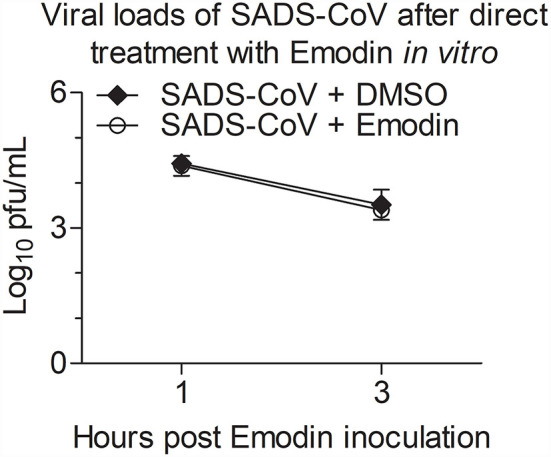
Emodin cannot directly impair SADS-CoV infectivity. SADS-CoV was co-incubated with Emodin (12.5 μg/ml) or 0.1% DMSO at 37 °C for 1 h or 3 h, and then viral titers were determined in Vero cells with the TCID_50_ assay. Results are representative of three independent experiments (mean ± SD). *n* = 3.

To determine whether Emodin has direct inactivation of SADS-CoV virions, Emodin (12.5 μg/ml) or 0.1% DMSO was co-incubated with the virions at 37 °C for 1 h and 3 h, and then the infectivity of the virions was detected by the TCID_50_ assay. As shown in Figure 3, the virus titers did not have a dramatic reduction in Emodin-treated groups as compared with the drug-free groups, indicating that Emodin can not directly impair SADS-CoV infectivity.

### Emodin acts at the whole phases of the SADS-CoV life cycle

**Figure 4:**
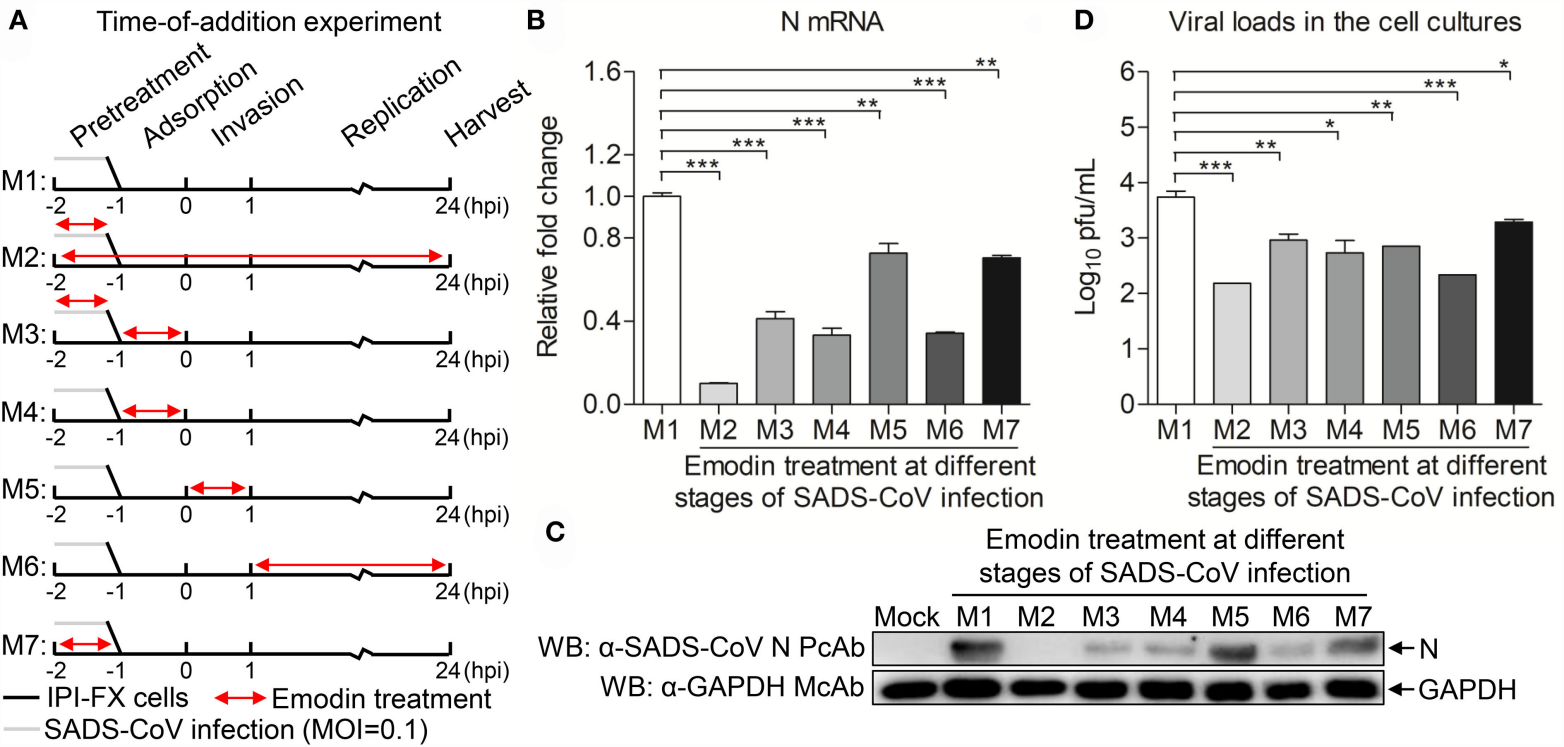
Emodin inhibits SADS-CoV infection at multiple steps of the virus life cycle. **(A)** SADS-CoV-infected IPI-FX cells were co-incubated with Emodin (12.5 μg/ml) or 0.1% DMSO at different stages of the viral replication cycle (M1–M7). Cell lysates were collected at 24 h after infection. **(B)** Real-time PCR was used to examine the mRNA expression of *N* and *GAPDH* using specific primers. The expression levels of mRNA were calculated in relation to the expression level of *GAPDH*. **(C)** The expression of N and GAPDH proteins was detected by Western blot using anti-SADS-CoV N polyclonal antibody and anti-GAPDH monoclonal antibody. **(D)** The viral titers were measured by the TCID_50_ assay. Data are representative of three independent experiments (mean ± SD). *n* = 3. *p < 0.05, **p < 0.01, ***p < 0.001.

To examine that Emodin blocks the stages of the SADS-CoV replication cycle, Emodin was directly added to co-incubate with the virus or IPI-FX cells at different phases of infection. As shown in Figure 4A, M1 represents the control group without Emodin treatment. M2 represents the cells and viruses treated with Emodin throughout the infection. M3 represents the viruses pretreated with Emodin, with Emodin added during the adsorption stage. M4 represents that Emodin was only added during the adsorption stage. M5 represents that Emodin was only added during the invasion phase. M6 represents that Emodin was only added during the replication phase. M7 represents cells pretreated with Emodin. Twenty-four hours after SADS-CoV infection, the cell samples were collected to test the mRNA and protein expression levels of SADS-CoV N and viral titers. Results are presented in Figures 4B–D. The mRNA and protein expression levels of N and virus titers all dropped significantly in the M2-M7 groups, indicating that Emodin has an anti-SADS-CoV effect on the whole phases of the SADS-CoV replication cycle.

### Emodin's anti-SADS-CoV activity might mainly involve with blocking viral attachment and activating the TLR3-IFN-λ3-ISG15 pathway

**Figure 5:**
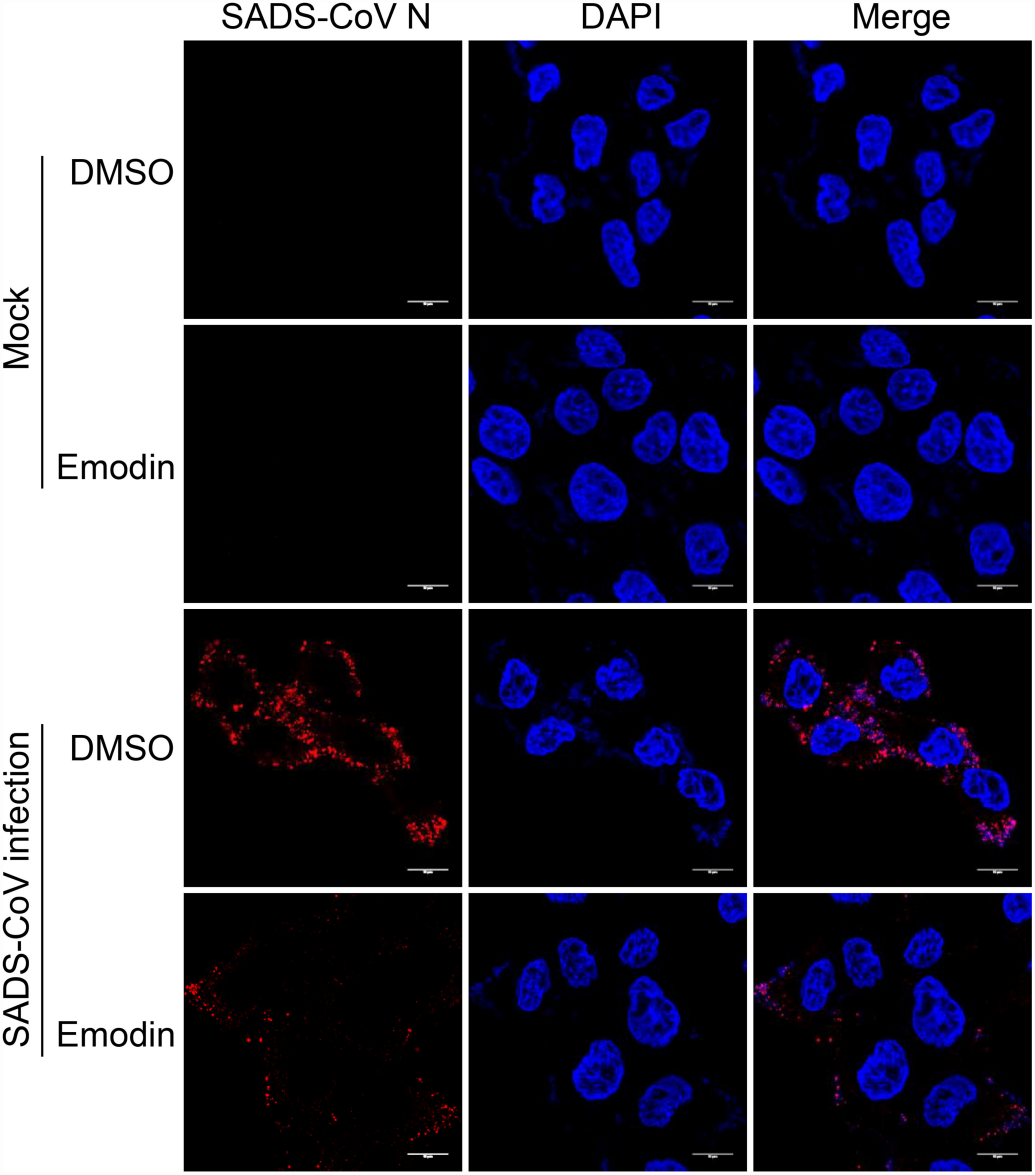
Emodin blocks the attachment of SADS-CoV to cells. IPI-FX cells were incubated with SADS-CoV together with Emodin (12.5 μg/ml) or 0.1% DMSO at 4 °C for 2 h. Images were taken using Leica TCS SP8 STED 3X microscopy with a 100 × NA oil-immersion objective. Scale bar, 10 μm.

Although Emodin acts on the entire phases of the SADS-CoV replication cycle, the antiviral effect is most significant in the viral attachment and replication stages. To further determine the effect of Emodin on virus adsorption, the viral attachment assay was carried out. As shown in Figure 5, Emodin can reduce virus particles attaching to the cell surface.

**Figure 6:**
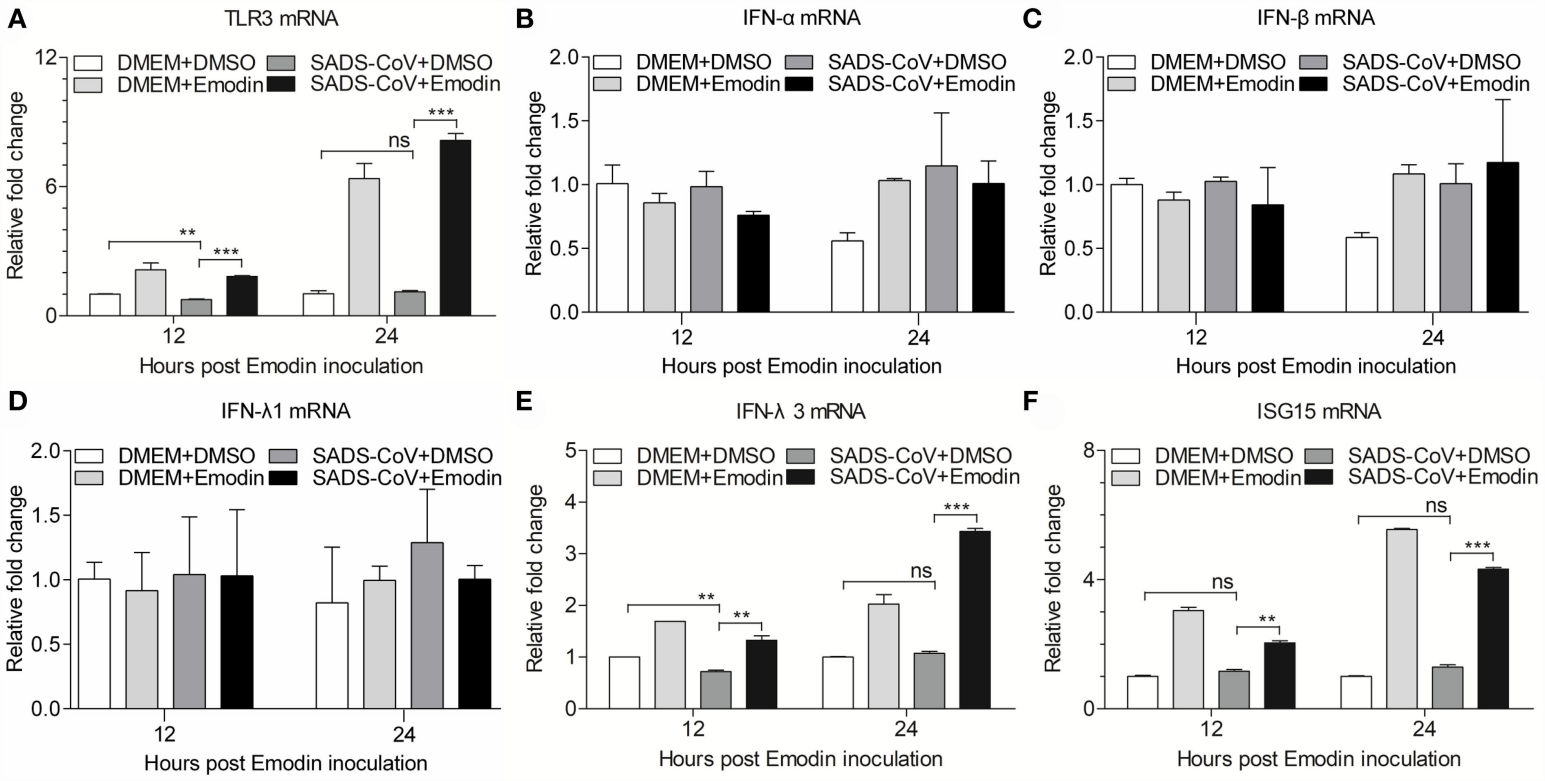
Emodin induces activation of TLR3-IFN-λ3-ISG15 pathway in SADS-CoV-infected cells. IPI-FX cells were incubated with Emodin (12.5 μg/ml) or 0.1% DMSO for 1 h, followed by infection with SADS-CoV at an MOI of 0.1. After 1.5 h, the cells were re-treated with Emodin (12.5 μg/ml) or 0.1% DMSO. DMEM + DMSO, DMEM + Emodin, and SADS-CoV + DMSO groups were as controls. At indicated time points (12 h and 24 h), the mRNA expression of *TLR3, IFN-*α, *IFN-*β, *IFN-*λ*1, IFN-*λ*3, ISG15*, and *GAPDH* was examined by quantitative real-time PCR using specific primers. **(A–F)** The expression levels of these molecules were calculated in relation to the mRNA expression level of *GAPDH*. Results are representative of three independent experiments (mean ± SD). *n* = 3. ** *p* < 0.01, *** *p* < 0.001.

Previous studies have confirmed that Emodin can promote the expression of type I IFN through the TLR3 pathway (17). To examine the expression of TLR3 and IFN-α/β in SADS-CoV-infected cells treated with Emodin, the mRNA expression levels of TLR3 and IFN-α/β were detected by qRT-PCR. We found that Emodin significantly increases the mRNA expression of TLR3 but did not affect the expression of IFN-α/β in SADS-CoV-infected IPI-FX cells (Figures 6A–C). It has been reported that gastrointestinal epithelial cells have a unique mechanism that can activate the type III IFN pathway through TLR3 to exert antiviral activity (32, 33). We further examined the mRNA expression of type III IFN and ISG15. As shown in Figures 6D–F, the mRNA expression of IFN-λ3 (*p* < *0.01*) and ISG15 (*p* < *0.01*) significantly increased in Emodin-treated infected cells as compared with the non-drug-treated infected cells, but the mRNA expression of IFN-λ1 had no significant change. These results suggested that the anti-SADS-CoV activity of Emodin may be due to blocking viral attachment and activating the TLR3-IFN-λ3-ISG15 pathway.

## Discussion

Since SADS-CoV has been reported in Guangdong of China in 2017 (1), this novel porcine enteric CoV was widely detected in southern China (8, 34), resulting in significant economic losses to pig farms. A recent study also confirmed that SADS-CoV can infect chickens and cause mild respiratory symptoms (35), indicating that it might also be a threat to the poultry industry. In addition, SADS-CoV also infected multiple human cell lines (10). These studies suggested that exploitation of the prevention and control measures for SADS has important significance to public health and livestock and poultry industries. In this study, we provided evidence that Emodin from Aloe could effectively inhibit SADS-CoV replication *in vitro*, which might help in the prevention and treatment of SADS-CoV infection.

In recent years, especially since the emergence of SARS-CoV-2 in 2019, more and more research has focused on anti-coronavirus drugs. As a novel coronavirus, there are no clinical records of anti-SADS-CoV drugs. Plants and plant-derived compounds have been a source of new antiviral drugs because of their advantages of low cost, few side effects, and high availabilities (36). Aloe vera is a perennial evergreen herb of the Liliaceae family, which is known for its immunomodulatory, anti-inflammatory, and antiviral properties (37, 38). Aloe vera exerts antiviral activity against multiple viruses, such as herpes simplex virus type 1, influenza virus, pigeon paramyxovirus type 1, and PRRSV (14, 15, 17, 39). Moreover, Aloe has been confirmed that it can inhibit PEDV infection *in vitro* and *in vivo* (16), which prompted us to test whether Aloe also has an inhibitory effect on SADS-CoV. In this study, we confirmed that Aloe has anti-SADS-CoV activity *in vitro*, indicating that Aloe has a broad-spectrum anti-coronaviruses property and can be used to screen for new antiviral drugs. However, whether Aloe can also resist SADS-CoV infection *in vivo* requires more research.

Of note, the Aloe used in this study, a water extract from the body of Aloe ferox, is crude, containing many good or bad components (17). To remove the harmful components and develop new generations of antiviral agents, it is necessary to determine the anti-SADS-CoV compounds in Aloe. Aloin, Quercetin, and Emodin were identified in Aloe in our previous study (17), and these three components have been previously reported to have antiviral effects (22, 40, 41), which prompted us to test whether these three components have anti-SADS-CoV activities. We found that Emodin showed the best anti-SADS-CoV effect among these three drugs (Figure 2). Emodin, an anthraquinone derivative, is known to possess several biological properties, including anti-bacterial, anti-inflammatory, antitumor, antivirus, and immunosuppressive properties (42). As is known to all, antiviral drug resistance to viruses is a key factor affecting the duration of antiviral drugs. When antiviral drugs are used, some viral particles survive from antiviral drugs, mutate, and accumulate resistance to the antiviral drugs (43). Compared to DNA viruses, RNA viruses are more likely to develop antiviral drug resistance based on their higher mutation rates (44). The potency of the antiviral drug is one of the key factors to the resistance of the antiviral agent (45). This study revealed that Emodin can inhibit SADS-CoV infection by targeting the adsorption, invasion, and replication stages of its replication cycle, and stimulating host innate immunity. In addition, we found that Emodin at 12.5 μg/ml is able to decrease SADS-CoV titers to undetectable levels and completely inhibits the viral replication at 12 hpi (Figure 2E), indicating that Emodin may be a potentially highly potent drug (45, 46), which suggested that the appropriate increased concentration of Emodin will help destroy SADS-CoV completely and reduce its resistance mutations (47). It has been reported that Emodin can inhibit SARS-CoV and HCoV-OC43 by blocking the S-ACE2 interaction and viral release, respectively (22, 23), indicating that Emodin can affect virus attachment and virus release. This phenomenon was also observed in Emodin suppression of SADS-CoV (Figures 4, 5). In addition, Emodin has effects on SADS-CoV invasion and replication (Figure 4), but the exact mechanism needs more studies in the future. Although Emodin could directly inhibit PRRSV infection in the absence of cells (17), this phenomenon had not been found in SADS-CoV (Figure 3), indicating that Emodin exerts its anti-SADS-CoV associated with host cells.

TLR3 is an intracellular pattern-recognition receptor that recognizes dsRNA to stimulate host antiviral immunity (29). Emodin inhibits PRRSV *via* TLR3 activation (17), prompting us to examine the effect of Emodin on the TLR3 pathway in SADS-CoV-infected IPI-FX cells. In the present study, we found that the mRNA expression of TLR3 (*p* < 0.001) was significantly increased in SADS-CoV-infected cells after Emodin treatment. On the contrary, the inhibition of coxsackievirus B3m infection by Emodin *via* downregulating the TLR3 pathway in BV2 cells (48) indicated that Emodin might have different antiviral mechanisms to inhibit different viruses. Once the receptor is activated, the downstream signal transduction is initiated to induce the expression of a variety of cytokines, including interferons (49). It has been reported that gastrointestinal epithelial cells can resist human enterovirus infection by activating the TLR3-IRF1-type III IFN axis (32, 33). Our results demonstrated that Emodin can increase the mRNA expression of IFN-λ3 (*p* < 0.01) in IPI-FX cells after SADS-CoV infection, which might be the result of TLR3 activation. Interestingly, Emodin could not induce IFN-α and IFN-β expression in IPI-FX cells but increased the expression of IFN-α and IFN-β in iPAMs (17), indicating that Emodin has different effects on different cells. It has been known that IFN-stimulated genes (ISGs) can be trigged after IFN production to exert antiviral activity (50), such as dsRNA activated protein kinase R (PKR) (51), 2′-5′-oligoadenylate synthetase (OAS) (52), and ISG15 (50). In this study, we found that Emodin could increase the mRNA expression of ISG15 (*p* < 0.01) but not PKR and OAS (data not shown) in IPI-FX cells, indicating that Emodin could activate the TLR3-IFN-λ3-ISG15 pathway. Since Emodin from Ae inhibited SADS-CoV infection mainly through blocking viral attachment and activating the TLR3-IFN-λ3-ISG15 pathway in cell culture, several important questions need to be addressed. For example, can Emodin inhibit SADS-CoV infection *in vivo*? What is the exact underlying mechanism of Emodin inhibits SADS-CoV? Elucidation of these questions will help us develop better strategies to prevent and control SADS-CoV.

Our results demonstrated that Emodin from Aloe inhibits SADS-CoV infection by blocking viral attachment and activating the TLR3-IFN-λ3-ISG15 pathway. Emodin might be utilized to prevent and control SADS-CoV infection.

## Data availability statement

The original contributions presented in the study are included in the article/Supplementary material, further inquiries can be directed to the corresponding author.

## Author contributions

ZX conceived and designed the experiments. ZX and SZ analyzed the data. SZ and ZX performed the experiments and wrote the manuscript. YC, ZX, CX, XW, HH, YX, XD, and WQ contributed reagents, materials and analysis tools. YC checked and finalized the manuscript. All authors read and approved the final manuscript.

## Funding

This work was supported by the Special Fund for Biosecurity Technology of Guangdong Province (2022B1111030001), National Key Research and Development Program, China (2021YFD1801101), and National Natural Science Foundation, China (#32172830 and #31902248).

## Conflict of interest

The authors declare that the research was conducted in the absence of any commercial or financial relationships that could be construed as a potential conflict of interest.

## Publisher's note

All claims expressed in this article are solely those of the authors and do not necessarily represent those of their affiliated organizations, or those of the publisher, the editors and the reviewers. Any product that may be evaluated in this article, or claim that may be made by its manufacturer, is not guaranteed or endorsed by the publisher.
