# Supplementary figures and images for: Emodin from Aloe inhibits Swine acute diarrhea syndrome coronavirus in cell culture
Source: Front Vet Sci. 2022 Aug 18;9:978453. doi: 10.3389/fvets.2022.978453 (PMC9433657; doi:10.3389/fvets.2022.978453)

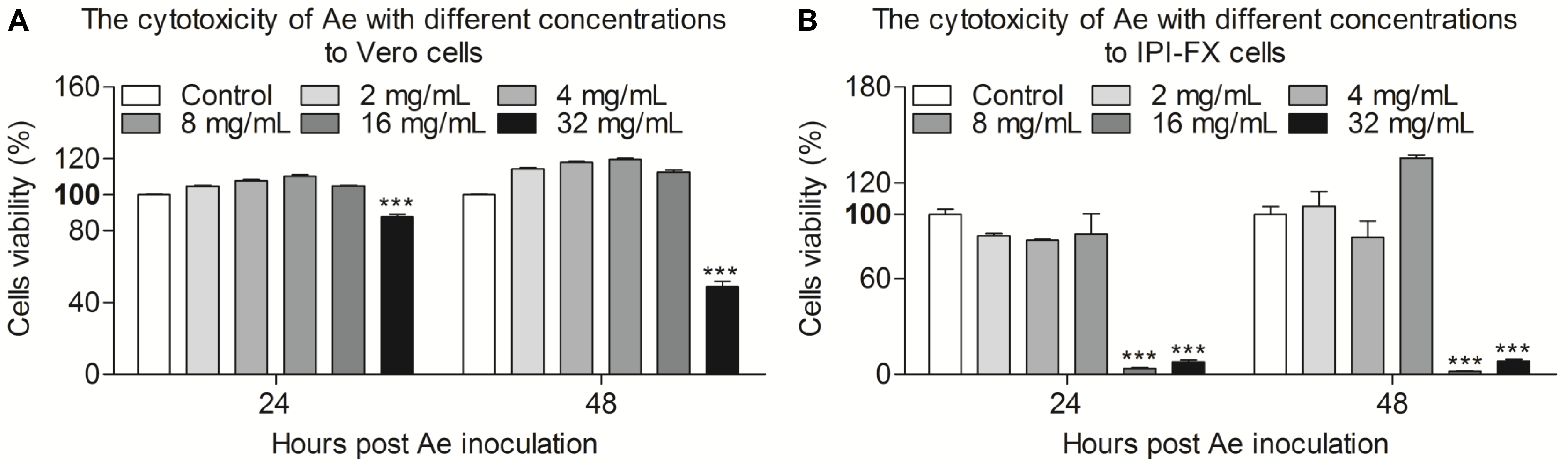

Supplement: Supplementary file 1 [file Image_1.TIF]
