# Supplementary material for: Emodin from Aloe inhibits Swine acute diarrhea syndrome coronavirus in cell culture
Source: Front Vet Sci. 2022 Aug 18;9:978453. doi: 10.3389/fvets.2022.978453 (PMC9433657; doi:10.3389/fvets.2022.978453)
Supplement: Supplementary file 2 [file Table_1.DOCX]

**Figure legend**

**Supplementary Fig.1. The cytotoxicity of Ae to Vero or IPI-FX cells.**

CCK-8 assay was used to detect the relative viability of Vero (A) or IPI-FX cells (B) co-incubated with different concentrations of Ae (2-32 mg/mL) or the control normal medium for 24 h and 48 h. Results are representative of three independent experiments (mean ± SD). n = 8. *** *p* < 0.001.
